# Sensitivity to Polymyxin B in El Tor *Vibrio cholerae* O1 Strain, Kolkata, India

**DOI:** 10.3201/eid2111.150762

**Published:** 2015-11

**Authors:** Prosenjit Samanta, Priyanka Ghosh, Goutam Chowdhury, Thandavarayan Ramamurthy, Asish K. Mukhopadhyay

**Affiliations:** National Institute of Cholera and Enteric Diseases, Kolkata, India

**Keywords:** *Vibrio cholerae* O1, cholera, vibrio infections, polymyxin B, bacteria, bacterial infections, antibacterial agents, pandemic, India

**To the Editor:** The epidemiology of cholera, especially in Africa and Asia, has periodically changed in subtle ways (*1*). The recent cholera epidemic in Haiti, a Caribbean country with no cholera cases in decades, affected >500,000 persons, caused ≈8,000 deaths, and brought this illness to the forefront of Haitian public health concerns (*2**,**3*). This life-threatening disease is caused by *Vibrio cholerae*, a waterborne bacterium with >200 serogroups, 2 of which, O1 and O139, cause epidemic or pandemic cholera. *V. cholerae* O1 is categorized as classical and El Tor biotypes, which differ biochemically and have different levels of virulence. Classical strains typically cause more severe illness than El Tor strains, which result in mild or moderate and sometimes asymptomatic cases. However, El Tor strains have replaced classical strains as the cause of cholera; the classical biotype is believed to be extinct, and El Tor strains currently prevail. However, the genetic traits specific to classical strains are still present in environmental and clinical *V. cholerae* isolates. Currently, all clinical strains of *V. cholerae* in Kolkata produce classical cholera toxin. Such phenotypic and genetic changes in *V. cholerae* are being monitored worldwide.

Several phenotypic and genetic laboratory tests are used to determine whether isolates are classified as classical or El Tor biotypes. Among phenotypic traits distinguishing the 2 biotypes, sensitivity to polymyxin B (50 U) is considered a reliable indicator and stable phenotype for biotyping. Research has shown that the genome of *V. cholerae* strains is undergoing cryptic changes that influence the strains’ virulence, rapid transmission, and spread (*4*). Our previous findings showed El Tor strains with few biotype traits of classical strains (*5*).

 Since the seventh cholera pandemic, which occurred during the 1960s and 1970s and was caused by El Tor strains, the El Tor biotype had been resistant to polymyxin B, a cationic antimicrobial peptide. However, when cholera strains first appeared in patients in Kolkata, India, in June 2012, *V. cholerae* O1 was found to be sensitive to polymyxin B (*6*). To determine whether this phenomenon occurred earlier, we tested 255 clinical strains isolated from patients in Kolkata during 2003–2014 and found that, from March 2013, polymyxin B–sensitive El Tor strains had replaced resistant strains (Figure, panel A). The MIC of polymyxin B, determined by Etest (bioMérieux, Marcy l’Etoile, France), confirmed that the El Tor strains were susceptible to this antimicrobial drug (Figure, panel B). In this assay, the El Tor strain (N16961) was highly resistant to polymyxin B (MIC 96 µg/mL), whereas the variant strains in Kolkata showed a drastic reduction in resistance (*6**,**7*).

**Figure:**
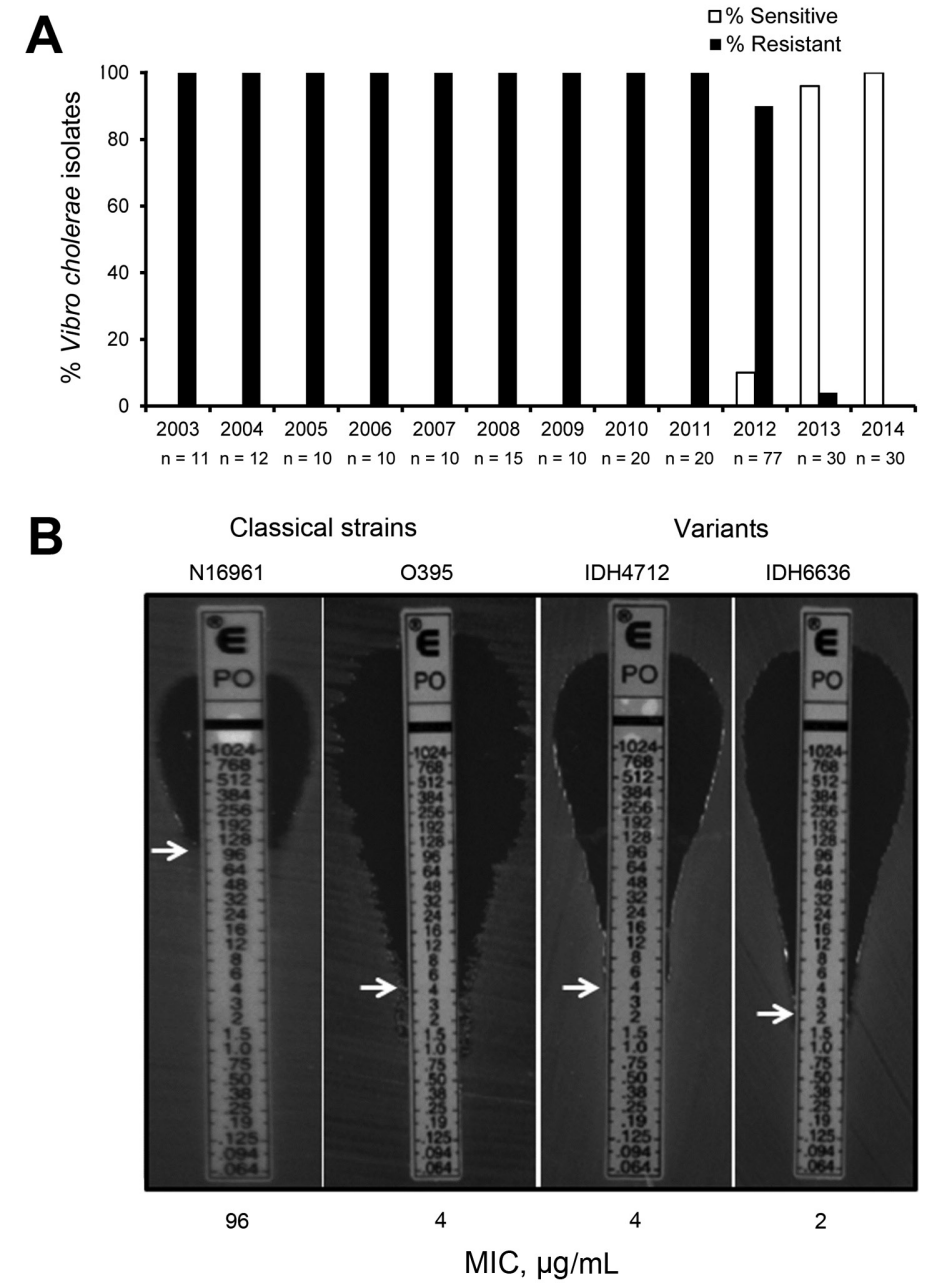
Isolation profile of polymyxin B–sensitive *Vibrio cholerae* strains in Kolkata, India, 2003–2014. A) Yearly occurrence of polymyxin B sensitivity and resistance in *V. cholerae* O1 El Tor variant strains isolated from Kolkata patients. During the study period, 255 strains were tested; n values indicate the number of strains tested each year. Polymyxin B–sensitive strains first appeared in Kolkata in June 2012. The first isolate in January 2013 was resistant, but, thereafter, all strains isolated during 2013–2014 were sensitive to polymyxin B, a biotyping marker for classical strains. B) MIC of polymyxin B in El Tor variant strains (classical and El Tor). MICs are indicated by white arrows. Polymyxin B sensitivity, a characteristic of classical strains, was displayed by El Tor variant strains. Data represent 3 biologic repetitions.

To confirm additional changes in biotype attributes in the variant Kolkata isolates during 2003–2014, we used the Voges-Proskauer test to determine production of acetylmethyl carbinol and found that the tested strains produced acetoin and were positive for chicken erythrocytes agglutination. The *rtsC* gene encoding the activator protein, which is absent from classical biotype strains but present in El Tor strains, was found in all the tested strains of the El Tor biotype. Biotype-specific CTX prophage repressor rstR was amplified with the El Tor–specific primers, indicating presence of El Tor rstR. The *tcpA* gene has distinct alleles specific to classical and El Tor biotypes of O1. Our study showed that all strains yielded amplicons with the El Tor-*tcpA*–specific primers but not with the classical-*tcpA*–specific primers. However, these strains had a single-base substitution at the 266-nt position of *tcpA*, also present in variant strains from Haiti. Furthermore, *Vibrio* seventh pandemic (VSP) gene clusters VSP I and VSP II are unique to El Tor strains of the seventh pandemic. We found presence of VSP I and II encoding genes in all our tested strains, indicating that the strains are El Tor, but with specific classical traits. 

We also checked the strains’ sensitivity to many antimicrobial drugs: tetracycline, trimethoprim/sulfamethoxazole, streptomycin, erythromycin, gentamicin, ciprofloxacin, and azithromycin, and all strains were sensitive to all drugs except trimethoprim/sulfamethoxazole and streptomycin. All strains isolated during 2013–2014 were fully resistant to trimethoprim/sulfamethoxazole and streptomycin, but 55% of strains isolated before 2012 were sensitive to these drugs.

Genes encoding lipid IVA acyltrasferase (*msbB*), biofilm formation, antimicrobial peptide resistance (*carR*), and 3 aminoacyl lipid modification (*almEFG*) have been shown to contribute to polymyxin resistance in *V. cholerae* (*6**–**8*). Analysis of these genes from the newly emerged polymyxin-B–sensitive strains may provide additional useful information. We found that these strains contained Haitian variant *ctxB* (*ctxB*7) similar to the classical cholera toxin. Our earlier studies identified many new attributes of Haitian *V. cholerae* variant strains in Kolkata since 2003 (*9**,**10*). 

We report the emergence of El Tor strains producing classical cholera toxin. These strains have lost an El Tor biotype marker and acquired a vital classical biotype characteristic, a change that has probably altered the regulatory mechanisms of lipid A modification machinery in *V. cholerae* (*6**–**8*). This change is a major event in the history of cholera after 1961, when El Tor strains first appeared. The recent changes in *V. cholerae* O1 strains should be carefully monitored to determine their clinical and epidemiologic implications.
